# Karma of Cardiovascular Disease Risk Factors for Prevention and Management of Major Cardiovascular Events in the Context of Acute Exacerbations of Chronic Obstructive Pulmonary Disease

**DOI:** 10.3389/fcvm.2019.00079

**Published:** 2019-06-25

**Authors:** Liliana Crisan, Nathan Wong, Don D. Sin, Hwa Mu Lee

**Affiliations:** ^1^Heart Disease Prevention Program, Division of Cardiology, University of California, Irvine, Irvine, CA, United States; ^2^Division of Respiratory Medicine, Department of Medicine, University of British Columbia and Centre for Heart Lung Innovation, Vancouver, BC, Canada; ^3^Division of Pulmonary and Critical Care Medicine, University of California, Irvine, Irvine, CA, United States

**Keywords:** chronic obstructive pulmonary disease, acute exacerbation of COPD, cardiovascular diseases, major adverse cardiovascular events, risk factors

## Abstract

There is compelling epidemiological evidence that airway exposure to cigarette smoke, air pollution particles, as well as bacterial and viral pathogens is strongly related to acute ischemic events. Over the years, there have been important animal and human studies that have provided experimental evidence to support a causal link. Studies show that patients with cardiovascular diseases (CVDs) or risk factors for CVD are more likely to have major adverse cardiovascular events (MACEs) after an acute exacerbation of chronic obstructive pulmonary disease (COPD), and patients with more severe COPD have higher cardiovascular mortality and morbidity than those with less severe COPD. The risk of MACEs in acute exacerbation of COPD is determined by the complex interactions between genetics, behavioral, metabolic, infectious, and environmental risk factors. To date, there are no guidelines regarding the prevention, screening, and management of the modifiable risk factors for MACEs in the context of COPD or COPD exacerbations, and there is insufficient CVD risk control in those with COPD. A deeper insight of the modifiable risk factors shared by CVD, COPD, and acute exacerbations of COPD may improve the strategies for reduction of MACEs in patients with COPD through vaccination, tight control of traditional CV risk factors and modifying lifestyle. This review summarizes the most recent studies regarding the pathophysiology and epidemiology of modifiable risk factors shared by CVD, COPD, and COPD exacerbations that could influence overall morbidity and mortality due to MACEs in patients with acute exacerbations of COPD.

Chronic obstructive coronary disease (COPD) is characterized by persistent respiratory symptoms and airflow limitation due to airway and/or alveolar abnormalities (1) and is recognized as a multicomponent disorder that can pre-dispose to many inflammatory complications. The most important systemic conditions associated with COPD are cardiovascular diseases (CVDs). Observational studies have shown that cardiovascular (CV) morbidity and mortality rates are more than double in the COPD cohorts compared with the general population (2, 3). They also found that during an acute exacerbation of COPD, patients are particularly vulnerable to major adverse cardiovascular events (MACEs) such as CV death, myocardial infarction (MI), stroke, heart failure, unstable angina, and transient ischemic attack (TIA). On the other hand, traditional CV risk factors such as smoking, hypertension (HTN), hyperlipidemia, diabetes mellitus (DM), air pollution, and physical inactivity are highly prevalent in patients with COPD, and they could contribute to COPD progression as well as aggravation of the MACEs. In the National Health and Nutrition Examination Survey (NHANES) study, adding global CV risk scores to COPD severity showed a 17.1% net reclassification improvement in predicting mortality in COPD patients due to CV events (4).

The exact mechanism by which COPD enhances risk of MACEs is not completely clear, but could be explained by airway and systemic inflammation resulting in over spilling of inflammatory cytokines in circulation leading to atherosclerosis. Also, COPD patients frequently demonstrate myocardial inflammation, arterial stiffness and endothelial dysfunction, intensified during acute exacerbations, possibly owing to “blood” inflammation and elastin degradation (5–7). Moreover, patients with COPD experience dynamic (and even static) hyperinflation, which may increase end diastolic pressures in the heart, and impair both right and left ventricular contractility that can further increase CV risk (8).

Nevertheless, there is evidence that a better control of CV risk factors can decrease probability of developing MACEs later in life, regardless of individual background, and it also can reduce COPD prevalence. According to our recent study with Life's Simple 7 scores in COPD, those with higher scores had better lung function and reduced COPD prevalence (9). Therefore, a better control of CV factors should be more emphasized in COPD patients as it could beneficially affect both CVD and COPD outcomes.

## Prevalence and Incidence

COPD is one of the few chronic diseases whose mortality and morbidity rates are increasing worldwide. According to the World Health Organization (WHO), in 2016 there were 251 million cases of COPD across the world (10). In the US, during 2014-2015, the prevalence of COPD was 5.9% (15.9 million) (11). Stratified by age groups, 2.1% of persons aged 18–24 years old were diagnosed with COPD, 2.5% of adults aged 25–34, 3.4% of adults aged 35–44 years old, 6.5% of those aged 45–55, 9.6% of those aged 55–64, and 12.5% of adults aged 65 or older (11). Data from a study of 6,266 individuals, who participated in the NHANES III (1988-1994), found that the prevalence of mild COPD was 12.1%, moderate 8.7%, and severe 1.7%, respectively (4). Despite the high prevalence of COPD, millions of people, especially during early or mild stages of COPD, are unaware of their disease. According to the US Department of Health and Human Services, ~50% of Americans who may have COPD remain undiagnosed (12). A systematic review found that the crude prevalence of CVD in COPD patients ranges from 28 to 70% (13). Depending on the study, the prevalence of ischemic heart disease in COPD patients ranges from 20 to 60%, heart failure 10–30%, arrhythmia 10–15%, and stroke ranges from 10 to more than 20% in hospitalized COPD patients (14). In the Study to Understand Mortality and Morbidity (SUMMIT), among 16,485 participants with FEV_1_ between 50 and 70% of predicted and high risk or history of CVD, but not severe heart failure, during 1.5 years follow-up, 28.5% of participants had at least one acute exacerbation of COPD and 4.2% of participants had at least one MACE. The highest incidence of MACEs, 26.7 per 100 patient-years, was noticed in the first 30 days after a hospitalized COPD exacerbation. Among those with MACEs, the CV deaths were noticed in 39.4% of patients, MI in 25.1% of patients, stroke in 18.5% of patients, unstable angina in 12% of patients, and TIA in 5% of patients (15). In another study, of the 25,857 patients with COPD who entered in the Health Improvement Network database, after COPD exacerbation, the incidence of MI was 1.1 per 100 patient-years, the risk for MI was increased by 2.27 times within 1–5 days, and the risk for stroke was increased by 1.26 times within 1–49 days (16). Rothnie et al. found that incidence rate ratio for MI was 2.58 for severe exacerbations of COPD and 1.58 for moderate exacerbations of COPD (17). In the Rotterdam study, among 13,471 individuals aged at least 45 years admitted in study, there were 12% of patients with COPD and 4.1% who suffered sudden cardiac deaths (SCDs). After 2,000 days of follow-up, the patients with COPD, compared to those without COPD, had a double risk of SCD, and the patients with frequent exacerbation of COPD had more than a triple risk of SCD (18).

## Risk Factors for MACEs in COPD

According to the most recent studies, COPD is an independent risk factor for CVD (19, 20). The acute exacerbations of COPD highly increase risk of MACEs. CV risk factors, which are well-established in inducing CVD and MACEs, are highly prevalent in COPD patients (15), and they also could contribute to COPD progression and exacerbations.

However, data on how risk factors can trigger MACEs after COPD exacerbation are limited. Van Eeden et al. describe some possible pathways by which risk factors that produce chronic lung inflammation could lead to CVD, and by which those that induce acute lung inflammation could lead to MACEs (21). According to their theoretical model, risk factors such as smoking, air pollution, and chronic infections could lead to lung inflammation mediated by macrophage and airway epithelial cells. The inflammatory mediators from lung tissue can flow in systemic circulation and lead to atheromatous plaque initiation, progression and destabilization, and CVD. In addition, an acute lung injury could further increase inflammatory markers, reactive oxidative species (ROS), and oxidized low-density lipoproteins (Ox-LDL), which lead to plaque destabilization as well as endothelial activation and dysfunction responsible for MACEs (increased endothelial permeability and endothelin-1, decreased vasodilatation and nitric oxide) (21).

Although multiple studies have tried to validate markers with clinical relevance in COPD patients, the results are inconsistent. However, according to a systematic review and meta-analysis, the shorter 6-min walk distance, high heart rate, fibrinogen, CRP, and white cell count were correlated with clinical outcome in patients with stable COPD (22). Another meta-analysis found that COPD exacerbation was constantly associated with increased CRP, and infrequently associated with IL6 and TNF-α (23). A systematic review noticed that fibrinogen, IL6, CRP, and total bilirubin are the most predictive markers of mortality in COPD mortality (24). Adamson et al. found that plasma cardiac Troponin I is a specific marker for future MACEs and CV deaths in patients with COPD and CV risk (25). Zagaceta et al. concluded that the most non-invasive marker that predicts MACEs in COPD is coronary calcium calcification (26). Bhatt et al. observed a positive correlation between coronary artery calcification, centrilobular emphysema, and increased plasma of CPR, fibrinogen, MMP3, CXCL9, and VCAM1, and decreased plasma CXCL5 (27).

**Figure 1:**
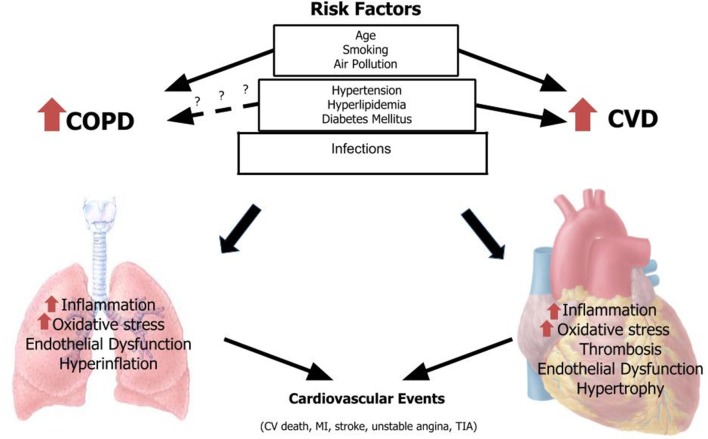
Risk factors for COPD and CVD associated with major cardiovascular events. CVD, cardiovascular disease; COPD, chronic obstructive pulmonary disease; MI, myocardial infarction; TIA, transient ischemic attack.

In the following article we will focus on epidemiology and mechanisms through acute exacerbation of COPD and specific risk factors common to CVD and COPD may increase risk of MACEs in patients with COPD (Figure 1; Tables 1–3).

**Table 1:** Pathophysiology of risk factors for CVD, COPD, and MACEs.

**Risk factors**	**Pathophysiology**
Acute exacerbation of COPD	Multifactorial (viruses, bacteria, smoking, air pollution, GERD)
	Initiates the inflammatory milieu
	Atheromatous plaque destabilization
	Induces arterial stiffness and myocardial inflammation
Smoking	COPD progression and exacerbation
	Atheromatous plaque initiation, progression, and destabilization
	Diminishes transcription factor FOXP3 with role in T cell development
	Reduces cilia, stimulates macrophages
	Promotes the hypercoagulation state, activates plateles
	Accentuates endothelial dysfunction
	Increases ox-LDL
	May also have anti-inflammatory effect
Air pollution	Atheromatous plaque initiation, progression, and destabilization
	Contibutes to COPD progression and exacerbation
	Increases inflammation
Hyperlipidemia	Atheromatous plaque initiation, progression, and destabilization
	Ox-LDL activates transcription factors involved in COPD pathogenesis
	Ox-LDL increases chemotaxis of neutrophils, eosinophils, and monocytes
	Ox-LDL increases pro-inflammatory cytokines
	OxLDL increases ROS production
Diabetes mellitus	Increases systemic inflammation and oxidative stress
	May cause direct damage by hyperglycemia
	Involved in myocelular hypertrophy and fibrosis
Hypertension	Induces structural alterations of the left ventricle and atrium
	Induces vascular system alterations
	Atheromatous plaque progression
	Increases ROS via NADPH oxidase stimulated by angiotensin II

*COPD, chronic obstructive disease; GERD, gastroesophageal reflux disease; Ox-LDL, oxidized low-density lipoprotein; ROS, radical oxygen species*.

**Table 2:** Evidence of risk factors that could contribute to MACEs.

**Risk factors**	**References**	**Study design**	**Findings**
COPD exacerbations	Kunisaki et al. (15)	Cohort analysis	3.8 HR for CVD events 30-day post-exacerbation
	Kunisaki et al. (15)	Cohort analysis	9.9 HR for CVD events 30-day post-hospitalized exacerbation
	Donaldson et al. (16)	Retrospective	2.27 higher MI risk 1 to 5 days post-exacerbation
	Donaldson et al. (16)	Retrospective	1.26 higher stroke risk 1 to 49 days post-exacerbation
	Rothnie et al. (17)	Retrospective	2.58 incidence rate ratio for MI, severe exacerbation
	Rothnie et al. (17)	Retrospective	1.58 incidence rate ratio for MI, moderate exacerbation
Smoking	Hackshaw et al. (28)	Meta-analysis	RR of CHD, 1 cigarrete/day: 1.74 for men, 2.19 for women
			RR of CHD, 20 cigarretes/day: 2:27 for men, 3.95 for women
	Center for disease control and prevention		Risk of CHD mortality: increased by 4 times for men, 5 times for women
			Risk of COPD mortality: increased by 17 times for men, 12 times for women
	Mulpuru et al. (29)	Prospective, multicenter	Smokers with COPD exacerbations: double risk for ICU admision
	Lubin et al. (30)	Prospective cohort	RR for CVD: higher with duration vs. qauntity smoked/day
	Bhatt et al. (31)	Cross-sectional, multicenter	RR for COPD: higher with duration vs. pack-years index
Air pollution	World Health Organization (32)		Causes 7 mil. deaths worlwide: 58% CVD and 18% COPD
	Pope et al. (33)	Prosective cohort	1.34 HR for CVD mortality with long-term exposure to PM2.5
Hyperlipidemia	Bartlett et al. (34)	Prosective cohort	1.3 OR for CVD with low HDL-C+TG or LDL ≥ 100 mg/dl
			1.6 OR for CVD with low HDL-C+TG and LDL-C ≥ 100 mg/dl
	Tipping et al. (35)	Meta-analysis	1.13 RR for CHD/3.5-fold higher usual Lp(a)
	Shen et al. (36)	Prospective cohort	Increased serum Ox-LDL in COPD patients vs. controls
	Basil et al. (37)	Cross-sectional	COPD vs. controls: no diference in TC, HDL-C, LDL-C and TG
			COPD patients: lower Apo B-100 and Lp(a)
	Chan et al. (38)	Retrospective	Hyperlipidemia+COPD: 0.64 HR for mortality
Diabetes mellitus	Baigent et al. (39)	Meta-analysis	Increases CVD mortality by 2–6 times
	Peters et al. (40)	Systematic review	CHD risk higher for women vs. men with DM
	Mulpuru et al. (29)	Prospective	COPD exacerbation+DM with end organ complication: 3.5 OR for ICU admission
			COPD exacerbation+DM without end organ complication: 1.7 OR for ICU admmission
Hypertension	Lawes et al. (41)		Causes 47% of all CHD and 54% of all stroke
	Lewington et al. (42)	Meta-analysis	Double CVD mortality with every 20 mmg over BP of 115/75

*COPD, chronic obstructive pulmonary disease; CVD, cardiovascular disease; CHD, coronary heart disease; LDL, low-density lipoprotein; HDL, high-density lipoprotein, HR, hazard ratio; RR, relative risk; WHO, World Health Organization*.

**Table 3:** Benefits of controlled risk factors for CVD, COPD, and MACEs.

**Prevention**	**Benefits**	**References**
Influenza vaccination	30% decreased risk of MACEs over 1 year	(43)
Pneumococcal vaccination	14% decreased risk of MACEs	(44)
Prophylactic antibiotherapy	33% reduction in frequency of COPD exacerbations/person/year	(45)
	No significant effects on severe adverse events or all cause-mortality	(45)
Smoking cessation	32% decreased risk of death or recurrent MI	(46)
	35% decreased risk of death or heart failure hospitalization	(46)
	37% at 1 year and 62% at 3 years decreased risk of CHD mortality	(47)
	16% decreased risk of COPD exacerbations after 5 years of cessation	(48)
	35% decreased risk of COPD exacerbations after 10 years of cessation	(48)
Improved cookstoves	36% decreased risk of COPD in women	(49)
LDL-C control	22% reduction in MACEs/1.0mmol/L LDL-C reduction	(39)
Tight glycemic control	42% decreased risk of any MACEs	(50)
	57% decreased risk of nonfatal MI, stroke, or death from CVD	(50)
Tight blood pressure control	50% decreased risk of heart failure	(51)
	30–40% decreased risk of stroke	(51)
	20–25% decreased risk of MI	(51)

*CVD, cardiovascular disease; COPD, chronic obstructive pulmonary disease; MACEs, major adverse cardiovascular events; CHD, coronary heart disease; MI, myocardial infarction; LDL, low-density lipoprotein*.

## Acute Exacerbation of COPD

Acute exacerbation of COPD seems to play the key role in initiating of MACEs in COPD patients. In the SUMMIT trial, the risk of MACEs was increased by ten times in the first 30 days after an acute exacerbation of COPD that required hospitalization (15). Although viral and bacterial respiratory infections account for 70–80% of COPD exacerbation (52), other risk factors are air pollution (53), low temperature (54), and gastroesophageal reflux disease (55). History of frequent exacerbations in the previous year, female sex, depression, smoking, high COPD grade, influenza virus detections rate, and low temperature are associated with developing future exacerbations (54, 56). According to a review, respiratory viruses are presented in 22–64% of COPD exacerbations, and dual viral-bacterial infection in 6.5–27% (57). A recent study found that the most common viruses associated with hospitalized COPD exacerbations were Influenza A (31%), Rhinovirus (24%), and respiratory syncytial virus A/B (17%) (58). The recent profile of bacterial infection in acute exacerbation of COPD is slightly variable geographically. The most isolated bacteria in a study from Spain were *Pseudomonas aeruginosa* (30.7%), *Streptococcus pneumoniae* (26.1%), *Enterobacteriaceae* (20.4%), *Haemophilus influenzae* (15.9%), and *M. catarrhalis* (6.8%) (59). The most identified bacteria in a study from Korea were *P. aeruginosa* (13.0%), *S. pneumoniae* (11.4%), and *H. influenzae* (5.3%) (60).

A characteristic feature of acute exacerbation of COPD is increasing pulmonary inflammation that can “spill out” into systemic circulation and trigger MACEs. Patel et al. noticed that COPD exacerbations, especially those caused by airway infection, were associated with acutely increased arterial stiffness related to inflammation that declined slowly over a more-than-5-week recovery period. They also found that arterial stiffness was higher in patients in stable state of COPD who had frequent exacerbations (6). Kwong et al. found that the incidence ratio for MI was higher in the first 7 days after laboratory-confirmed respiratory viral infection (61), although other studies noticed that depending on infection severity, the risk of MI can be increased for a period of months, even up to 10 years, especially after respiratory infections (62). Post-mortem studies in humans observed that the atherosclerotic coronary arteries displayed more inflammatory cells after an acute systemic infection (63). Jaw et al. established a novel murine model of atheromatous plaque destabilization by administering lipopolysaccharide directly into the lungs of male mice, which reproduced an acute infection with Gram-negative bacteria (64). The signs of plaque destabilization such as hemorrhage and thrombus formation occurred as early 8 h in 68% of mice exposed to lipopolysaccharide and only in 12% of mice exposed to saline. It was also noticed that plaque vulnerability and rupture was prevented if the mice were depleted of circulating neutrophils before lipopolysaccharide administration, which revealed the key role of neutrophil from lung injury in pathogenesis of acute atheromatous plaque rupture (64).

A proven strategy to prevent severe COPD exacerbations and MACEs is influenza vaccination. A national multicenter prospective cohort study using data from the Canadian Immunization Research Network Serious Outcomes Surveillance (SOS) found that, among patients with COPD, vaccinated individuals compared to unvaccinated patients have a 38% reduction in influenza-related hospitalizations (29). In a randomized control trial lead by Phrommintikul et al. a 30 % decrease of MACEs over 1 year in patients with coronary diseases who were treated with inactivated influenza vaccine was observed (43). Also, pneumococcal vaccination can decrease the risk of CV events by 14% according to a meta-analysis; the best results were observed especially in the elderly with CV and pulmonary diseases, especially in the first year after vaccination (44). A review of 14 studies that included participants aged between 65 and 72 years, with moderate-severe COPD, found that the prophylactic antibiotic treatment decreased the frequency of COPD exacerbation/person/year by 33% (rate ratio 0.67; 95% CI 0.54–0.83), and continuous or intermittent antibiotic use may be more efficient than pulsed therapy. However, prophylactic antibiotics did not have a significant effect on frequency of hospitalization, serious adverse events, all-cause mortality, or the loss of lung function during the study period (three to 36 months) (45).

## Evidence for Specific Risk Factors Common to CVD and COPD

### Cigarette Smoking

According to the most recent data from Center for Diseases Control and Prevention, the risk of coronary heart disease (CHD) mortality due to smoking is increased by almost four times for middle-aged men, and by almost five times for women. The risk of dying from COPD due to smoking is increased by 17 times for men, and by 12 times for women. From 2005-2009 period, among smokers aged 35 years and older, the annual cigarette-smoking related mortality in the U.S. was 151,000 for CVDs and 100,600 for COPD (65). Current smoking status increased by 2 times the risk of ICU admission of the patients with COPD exacerbations due to influenza in the Canadian study mentioned above that used the SOS database (29). According to a meta-analysis of 141 cohort studies, women have a higher risk of CHD with smoking than men. After adjustment for multiple confounders, the relative risk (RR) of CHD was 1.74 for men who smoked one cigarette per day, and 2.27 for men who smoked 20 cigarettes per day. Among women, the RR of CHD was 2.19 for smoking one cigarette per day and 3.95 for smoking 20 cigarettes per day (28). The risk of CVD and COPD is higher with duration of smoking than with number of cigarettes smoked per day and the composite index of pack-years (30, 31).

More than 7,000 chemical compounds (USDHHS, 2010) are found in cigarettes that can affect the immune system. These include direct carcinogens, toxins [e.g., carbon monoxide [CO], ammonia, acetone, nicotine, and hydroquinone], reactive solids with chemically catalytic surfaces, and oxidants (e.g., superoxide and nitrogen oxides) (66). Smoking diminishes forkhead box PC (FOXP3), a transcription factor essential to the development of competent regulatory T cells in human airways (67). Lungs of smokers have reduced cilia, stimulated epithelial cells and macrophages, and increased neutrophils (68). Also, cigarette smoking promotes hypercoagulable states and narrowing of the vascular lumen that increase the risk of acute thrombosis (69). Oxidants contained in cigarettes contribute to atherosclerotic plaque formation by accentuating endothelial dysfunction, oxidizing of low-density lipoproteins (LDL) and activating platelets (70). During the periods of acute exacerbation of COPD, inflammation could induce plaque rupture, and MACEs. In spite of the pro-inflammatory effect of smoking, smoking can also induce (paradoxical) anti-inflammatory effects owing to its oxidant, CO, nicotine, and some aromatic contents that can modify transcriptional programs and extracellular matrix (66).

Many studies assessed the benefits of sustained smoking cessation on CVD and COPD, but is not clear how smoking cessation can affect the risk of MACEs after COPD exacerbations. The Survival and Ventricular Enlargement (SAVE) trial, which analyzed the risk of recurrent events after MI, has revealed at the 6-month follow-up that smoking cessation was associated with a 43% decrease in all-cause mortality, a 32% decrease in the risk of death or recurrent MI, and a 35% decrease in the risk of death or heart failure hospitalization (46). In the Multiple Risk Factor Intervention Trial (MRFIT), the relative risk of death from CHD was decreased by one-third in smokers who quit for 1 year and by almost two-thirds in those who quit for 3 years (47). Although some studies noticed not only decreased but also increased hospitalization in COPD patients who quit smoking for <5 years (71), smoking cessation may improve the pulmonary function, even in intermittent quitters (72), and can decrease the risk of acute exacerbations of COPD over time. A study of 23,971 veterans with COPD from the US has shown that the risk of COPD exacerbations was not affected in the first 5 years of smoking cessation, but it was decreased by 16% after quitting for 5–10 years, and by 35% for those who had quit for longer than 10 years (48).

### Air Pollution

Recent studies have shown that exposure to small particulate matter of 2.5 microns or less in diameter (PM2.5) and ozone, may have destructive effects in both the lung and heart, consequently increasing mortality due to cardiovascular and respiratory causes. The WHO estimates that in 2016 ambient air pollution caused 7 million pre-mature deaths worldwide, with 58% of deaths being related to ischemic heart disease and strokes, while 18% of deaths were due to COPD (32). A study across China concluded that over 1.3 billion people have high health risks associated with exposure to ambient fine particulate matter (PM2.5) (73). A representative cohort of the adult U.S. population from National Health Interview Survey (NHIS) data has found that after adjustment for individual risk factors, including smoking, the risk for CVD mortality was 34% higher with 10-μg/m^3^ exposure increment of PM2.5 (33).

However, the mechanisms of how air pollutants raise CVD and COPD mortality are not very well-elucidated. Experimental models noticed that air pollution causes an inflammatory process in peripheral airways and alveoli as well as atherosclerotic plaques progression in coronary artery. Mills et al. noticed that men with stable ischemic heart disease exposed to dilute diesel exhaust for 1 h during exercise had a considerable elevation in neutrophils, mast cells, CD4+, and CD8+ T lymphocytes as well as an increase expression of endothelial adhesion molecules ICAM-1, VCAM-1, and LFA-1 in bronchial tissue at 6 hours after exposure (74). The rabbits exposed to particulate matter even showed a positive correlation between atherosclerotic plaque volume, plaque vulnerability as well as systemic and lung inflammation (75). Kido et al. noticed a systemic inflammatory process and acute endothelial dysfunction of blood vessels in mice exposed to particulate matter. The integrity of the vascular endothelium was established after removing IL-6 that is an inflammatory mediator (76).

However, there are studies that show that the measures that prevent air pollution can also reduce COPD prevalence. A systematic review and meta-analysis of experimental studies revealed that improved cookstoves may decrease the risk of COPD in women by 26% (49).

### Hyperlipidemia

Numerous studies found that high levels of LDL-C and/or low levels of high-density lipoprotein-cholesterol (HDL-C) increase the risk of coronary atherosclerosis by enhancing endothelial dysfunction and inflammatory pathways in vascular endothelium. Recently, there is increasing evidence that lipoprotein (a) [Lp(a)] (77), as well as high triglycerides in low density-lipoprotein (LDL-TG) (78), are independently associated with increased incidence of CVD and that non-HDL-C is a better indicator of CVD risk than LDL-C, especially in patients with hypertriglyceridemia (79). The Framingham Heart Study Offspring Cohort has concluded that after adjustment for covariates, the odds of having CVD was 30% higher when low HDL-C was accompanied by either higher TG or higher LDL-C, and 60% higher when both TG and LDL-C levels are elevated (34). According to an analysis of 36 retrospective studies, the risk of CVD is also increased by 13% for each 3.5-fold increase in Lp(a) (35).

While there is a well-established relationship between hyperlipidemia and CVDs, the relationship between hyperlipidemia and COPD is not as well-established. One study found that among 48 patients with COPD and 32 controls, mean serum levels of ox-LDL were significantly higher in COPD patients, especially in those with GOLD Stage IV (18.62 ± 7.56 mU/L) than in controls (12.57 ± 5.90 mU/L, *P* = 0.05) (36). A cross-sectional study on 100 subjects found that patients with severe and very severe COPD had increased level of blood cholesterol and LDL, as well as low HDL. The highest levels levels of cholesterol (6.16 ± 1.5 vs. 5.61 ± 1.1, *p* = 0.039) were noticed in patients with very severe COPD (80). In contrast, Basil et al., in a study on 90 patients with COPD and 90 controls, did not find a significant difference of HDL-C, LDL-C, and TG between COPD patients and controls, and surprisingly, COPD patients had lower serum levels of Apo B-100 (*P* < 0.0001) and Lp(a) (*P* < 0.003) (37). Moreover, a retrospective study of 2,982 subjects, using the Taiwan National Health Insurance database found that among patients with COPD, hyperlipidemia was associated with 36% decreased incidence of mortality (38).

However, recent studies indicate that lipids can contribute not only to pathogenesis of CVD, but also of COPD. Ox-LDL stimulates numerous transcription factors with role in atherogenesis as well as COPD pathogenesis, increases ROS production and pro-inflammatory cytokines, and potentiates atherogenesis by penetrating vascular intima where increases the chemotaxis of neutrophils, eosinophils and monocytes (36, 81, 82). Vascular cell adhesion molecules 1 (VCAM-1) and intracellular adhesion molecule 1 (ICAM-1) stimulated by modified lipoproteins facilitate the transendothelial migration and attachment of inflammatory cells to endothelial cells (83). Like LDL, triglyceride-rich lipoproteins (TRL) potentiates atherogenesis by penetrating arterial intima and formation of fibrofatty lesions. But in contrast to LDL, TRL can be taken directly by arterial macrophage without previous oxidation (84). When plaque is ruptured, the lipids are extruded into the arterial lumen, inducing vasospasm and thrombus formation. Studies suggest that in COPD pathogenesis could also be involved lipid alterations such as promotion of fatty acid oxidation pathway, high levels of cholesterol or oxidizing cholesterol, an increased apoptosis of pulmonary epithelial cells induced by upregulation of ceramide, a dysregulated lipid metabolism of macrophages that decreases phagocytosis, and high levels of catabolic lipid mediators (85).

Nevertheless, there is ample evidence to indicate that improved control of hyperlipidemia reduces the risk of CVD, although it is not clear if tight lipid control could improve COPD evolution and decrease the risk of MACEs after COPD exacerbations. A meta-analyses of 26 randomized trials has shown that a 1.0 mmol/L reduction in LDL-C was associated at the 1-year follow-up with a 24% reduction of major coronary events and a 15% reduction of stroke. The greater benefits were observed with a higher reduction of LDL-C, even down to 1–2 mmol/L (39). Moreover, genetic mutations noticed a bigger reduction in CVD risk with a reduced lifelong LDL-C. The incident of CHD was decreased by 88% for mutations that decreased LDL-C by around 1 mmol/L (40 mg/dL), and by 50% for mutations that reduced LDL-C by 0.5 mmol/L (20 mg/dL) (86).

### Diabetes Mellitus (DM)

Abundant studies have revealed that patients with DM have two to six times higher risk of deaths from CVD compared to persons without DM (87). On average, CVDs are responsible for 52% of deaths in patients with type 2 diabetes mellitus (T2D) and 44% of deaths in patients type 1 diabetes, with a difference between sexes (88). A meta-analysis of 64 cohorts revealed that compared to men, women with DM have a 44 % higher risk of CHD, and a 27% higher risk of stroke (40, 89). DM may also be an independent risk factor for COPD. The Fremantle Diabetes Study, where 125 patients with T2D without history of lung disease, were evaluated by spirometry at baseline and followed for ~7 years, has found that the FEV_1_ was reduced in average by 71 mL/year for patients with T2D, although usually, FEV_1_ decreased by 25–30 mL/year in healthy non-smokers (90). The SOS study noticed that among the patients with COPD exacerbation due to influenza, the risk of ICU admission was increased by 3.5-fold for patients who had associated DM with end-organ complications and by 1.7-fold for patients with DM without end-organ complications (29).

Many studies have concluded that DM can cause biochemical changes in the structures of lung and heart tissue due to systemic inflammation, oxidative stress, or from the direct damage caused by chronic hyperglycemia. Accumulation of advanced glycation end products in the heart of patients with DM may cause myocellular hypertrophy and myocardial fibrosis from diabetic cardiomyopathy (91). Also, high blood and tissue concentrations of glucose may increase the responsiveness of airway smooth muscle cells to contractile agents, due to increased activation of the Rho-kinase pathway that mediates myosin-actin contractility, which in turn may contribute to accelerate the decline of FEV_1_ (92, 93).

The Diabetes Control and Complications Trial (DCCT) has noticed that for patients with type 1 DM, a tight glycemic control significantly decreased the risk of any MACEs by 42% and the risk of non-fatal MI, stroke, or death from CVD by 57% (50). In contrast, a retrospective case-control study using a long-established English general practice network database (*n* = 894,646) has found that over 8 years follow-up period, people with T2D had an 11% reduced risk of COPD vs. those without T2D (HR 0.89, 95%CI 0.79–0.93) (94). However, the benefits of tight glycemic control on MACEs after COPD exacerbation are not very well-documented.

### Hypertension (HTN)

HTN is the most important risk factor for CVDs, causing about 54% of all strokes and 47% of all CHD events across the world (41). A meta-analysis of 61 prospective observational studies of blood pressure has noticed that in blood pressure higher than 115/75 mmHg, for every 20 mmHg systolic or 10 mmHg diastolic increase in blood pressure, there is a doubling of CVD mortality (42). Also, there are studies that have shown that HTN is highly associated with COPD. For example, 90% of COPD participants with risk factors or history of CVD in the SUMMIT trial had HTN (15).

Hypertension may induce structural alteration of the left atrium and ventricle as well as of the vascular system and contribute to atheromatous plaque progression (95). Also, patients with COPD have an increased arterial stiffness that has been attributed to protease-antiprotease imbalance (responsible for emphysematous COPD), accelerated elastin degradation, systemic inflammation, altered redox balance, advanced glycation end products, and activation of renin-angiotensin-aldosterone-system (96, 97). Increased angiotensin II not only that contributes to vasoconstriction, heart hypertrophy, inflammation and fibrosis, but also may affect the lung by stimulating NADPH oxidase to generate ROS (98, 99).

According to several clinical trials, tight blood pressure control may reduce by 50% the risk for heart failure, by 35–40% for stroke, and by 20–25% for MI (51). The SPRINT trial found that patients with high CVD risk, but without diabetes, had a more accentuated reduction in the rates for fatal and non-fatal major cardiovascular events and deaths from any causes when they had a blood pressure of <120 mmHg, compared to those who had <140 mmHg (100, 101). However, further studies are needed for revealing the benefits of low blood pressure in the prevention of MACEs in patients with COPD.

### Physical Activity

There are observational studies that have noticed an important increase in hospitalization and/or mortality rates in COPD patients with a low level of physical activity (102, 103). Early supervised pulmonary rehabilitation could be an effective intervention for the reduction of mortality following hospitalization for an AECOPD. A meta-analysis has found that early rehabilitation in COPD patients was associated with a 42% relative risk reduction in mortality, 53% risk reduction in hospital readmission related to COPD, and a shorter period of hospitalization by 4.27 days (104).

## Medication Issues

Although the beneficial effects of beta-blockers are well-established in CVD, their benefit in COPD is uncertain. The underuse of β-blockers in patients with COPD may be an important factor by which COPD is associated with a residual risk of MACEs (4). Recently published reviews report an association between the use of β-blockers and improved survival in COPD patients with heart failure, MI, CVD, and HTN. A meta-analysis of fifteen observational cohort studies with a follow-up time from 1 to 7.5 years has revealed that beta-blocker treatment significantly reduced the relative risk (RR) for overall mortality by 28%, and for COPD exacerbations by 37%. For patients who had CHD or heart failure associated with COPD, the risk for overall mortality was decreased by 36 and 26% respectively (105). Dransfield et al., who examined data from 16,485 participants in the SUMMIT study have shown that baseline β-blocker therapy does not reduce the respiratory benefits of inhaled bronchodilators, or increase the cardiovascular risk of inhaled long-acting β-agonists in patients with COPD, who also had and heightened cardiovascular risk (106).

Recent research has increased the awareness of the initiation of the treatment with long-acting inhaled β-agonists and anticholinergics in patients with COPD. Gershon et al., conducted a nested case-control analysis of a retrospective cohort study on 191,005 patients from Ontario Health care database and found a 17% increased risk of a MACEs with the incidentally use of long-acting inhaled β-agonists and anticholinergics compared with non-use of those medications (107). In a meta-analysis, inhaled anticholinergics were associated with statistically significant increased the relative risk of MI by 52% and of cardiovascular death by 92%, without a statistically significant increase in the risk of stroke (108). Another meta-analysis that compared tiotropium Soft Mist Inhaler, tiotropium HandiHaler, long-acting β2 agonists (LABAs), inhaled corticosteroids (ICS), and LABA-ICS has found that only tiotropium Soft Mist Inhaler had an evident risk of CVD deaths, especially in patients with severe COPD (109).

Since hyperlipidemia is an independent risk factor for CVD, and since many patients with COPD die of CVDs, statins could have benefit in reducing mortality. Some authors suggest that statins, in addition to decreasing of serum cholesterol, may have anti-inflammatory effects on both pulmonary and systemic inflammation through inhibition of guanosine triphosphates and nuclear factor-κB that controls activation of inflammatory and matrix remodeling pathways (110). A meta-analysis of 15 articles with a total of 238,459 participants with COPD found that the use of statins was associated with a 31% reduced risk of MI (HR 0.69, 95% CI 0.49–0.99), but not of CVD mortality (111). Furthermore, this meta-analysis found a protective effect of statin treatment on the risk of COPD exacerbation with or without hospitalization, decreasing the risk by 36% (HR 0.64, 95% CI 0.55–0.75). This is in contrast with the randomized, controlled STATCOPE trial, published in 2014, involving almost 900 patients with COPD, but no patients with CVD, diabetes or those who were taking statins. The study showed no benefits of daily simvastatin treatment for reducing COPD exacerbation rates, or time to first exacerbation in high-risk patients (112).

## Strengths and Limitations

The main strength of this review is the comprehensive review of mostly observational and some limited clinical trial evidence on the relation different risk factors have both on CVD and COPD as well as the synergic effect of those risk factors with the acute exacerbation of COPD in initiating MACEs. We have also discussed putative mechanisms explaining how certain shared risk factors may also affect COPD. One limitation is the lack of intervention trials testing individual or composite risk factor interventions on CVD outcomes specifically in COPD patients.

## Future Directions

Research on the association and impact of shared risk factors between COPD and CVD is crucial because currently there are no proven methods of COPD prevention, except for smoking cessation. More studies are required to see if a tighter control of traditional CV risk factors may reduce MACEs in patients with COPD. If this is the case, we may consider specific targets and strategies that would apply for COPD patients with respect to control of blood pressure, HgA1c, and LDL. Tight glycemic control of diabetic patients with COPD may be even more important than the treatment of hyperlipidemia or hypertension control in reducing vulnerable plaque rupture following an episode of acute exacerbation of COPD. Recently, it was found that systemic inflammation plays a role in connecting all of these conditions together. Thus, we may be able to better manage morbidity and mortality in COPD patients by treating the composite risk factors shared by these conditions. Because there are no known ways to cure or reverse COPD, early screening and education on modifiable risk factors is essential in reducing the burden of COPD.

## Conclusions

Behavioral, metabolic, infectious and environmental risk factors are involved in the pathogenesis of both, COPD and CVD, and may help explain the increased risk of MACEs in COPD patients. There is a lack of prognostic predictors for COPD, except for spirometry measure and symptoms. Spirometry, in particular, is largely under prescribed. Missed diagnosis and severity stratification, combined with incomplete knowledge of adverse drug events lead to under-treatment of patients with both COPD and CVD. Moreover, pulmonologists do not typically perform appropriate CVD risk assessment in their COPD patients. Greater investigation and tight control of the risk factors that are responsible for many biochemical-physiological processes involved in CVD and COPD may result in the development of guidelines for therapeutic interventions aimed at decreasing MACEs after COPD exacerbations. There is an urgent need to consider methods to prevent these events by modifying CVD risk factors, and also changing lifestyle. We have previously demonstrated a strong association of global risk scores with the severity of COPD, and that their use improves risk stratification of patients with COPD. Also, the use of global risk scores such as the ASCVD Pooled Cohort Risk Calculator can be applied to determine their 10-year and lifetime risk of CVD and who may benefit from appropriate preventive therapies of CVD. We need to find out whether more tight control of risk factors in COPD may improve CVD outcomes. Thus, the use of appropriate CVD risk assessment in patients with COPD is important for identifying who may need appropriate CVD preventive therapies to prevent CVD death following exacerbation related sudden death and for predicting the long term survival of COPD patients.

## Author Contributions

LC, NW, and HL: substantial contributions to the conception or design of the work or the acquisition, analysis, or interpretation of data for the work. LC, NW, HL, and DS: drafting the work or revising it critically for important intellectual content. LC and NW: agree to be accountable for all aspects of the work in ensuring that questions related to the accuracy or integrity of any part of the work are appropriately investigated and resolved. NW, HL, and DS: provide approval for publication of the content.

### Conflict of Interest Statement

The authors declare that the research was conducted in the absence of any commercial or financial relationships that could be construed as a potential conflict of interest.

## References

[1] Global Initiative for Chronic Obstructive Lung Disease Pocket Guide to COPD Diagnosis, Management and Prevention. (2019). Available online at: https://goldcopd.org/wp-content/uploads/2018/11/GOLD-2019-POCKET-GUIDE (accessed May 27, 2019).

[2] Chen W Thomas J Sadatsafavi M FitzGerald JM. Risk of cardiovascular comorbidity in patients with chronic obstructive pulmonary disease: a systematic review and meta-analysis. Lancet Respir Med. (2015) 3:631–9. 10.1016/S2213-2600(15)00241-6 26208998

[3] Huiart L Ernst P Suissa S. Cardiovascular morbidity and mortality in COPD. Chest. (2005) 128:2640–6. 10.1378/chest.128.4.2640 16236937

[4] Lee HM Lee J Lee K Luo Y Sin DD Wong ND. Relation between COPD severity and global cardiovascular risk in US adults. Chest. (2012) 142:1118–112. 10.1378/chest.11-2421 22518027

[5] Paulus W J Tschope C. A novel paradigm for heart failure with preserved ejection fraction: comorbidities drive myocardial dysfunction and remodeling through coronary microvascular endothelial inflammation. J Am Coll Cardiol. (2013) 62:263–71. 10.1016/j.jacc.2013.02.092 23684677

[6] Patel AR Kowlessar BS Donaldson GC Mackay AJ Singh R George SN. Cardiovascular risk, myocardial injury, and exacerbations of chronic obstructive pulmonary disease. Am J Respir Crit Care Med. (2013) 188:1091–9. 10.1164/rccm.201306-1170OC 24033321 PMC3863745

[7] Luehrs RE Newell JD Comellas AP Hoffman EA Warner K Croghan A CT-measured lung air-trapping is associated with higher carotid artery stiffness in individuals with chronic obstructive pulmonary disease. J Appl Physiol. (2018) 125:1760–6. 10.1152/japplphysiol.00580.2018 PMC6859127 30307820

[8] Smith BM Prince MR Hoffman EA Bluemke DA Liu CY Rabinowitz D Impaired left ventricular filling in COPD and emphysema: is it the heart or the lungs? Chest. (2013) 144:1143–51. 10.1378/chest.13-0183 23764937 PMC3787914

[9] Fan W Lee H Lee A Kieu C Wong ND. Association of lung function and chronic obstructive pulmonary disease with american heart association's life's simple 7 cardiovascular health metrics. Respir Med. (2017) 131:85–93. 10.1016/j.rmed.2017.08.001 28947048

[10] World Health Organization. *Chronic Obstructive Pulmonary Disease*. Keys Facts. (2017) Available online at: https://www.who.int/news-room/fact-sheets/detail/chronic-obstructive-pulmonary-disease (accessed May 27, 2019).

[11] Sullivan J Pravosud V Mannino DM Siegel K Choate R Sullivan T. National and state estimates of COPD morbidity and mortality-United States, 2014-2015. Chronic Obstr Pulm Dis. (2018) 5:324–33. 10.15326/jcopdf.5.4.2018.0157 30723788 PMC6361472

[12] US Department of Health and Human Services *Research Portfolio Online Reporting Tools* Chronic Obstructive Pulmonary Disease. (2018). Available online at: https://report.nih.gov/nihfactsheets/ViewFactSheet.aspx?csid=77 (accessed May 27, 2019).

[13] Müllerova H Agusti A Erqou S Mapel DW. Cardiovascular comorbidity in COPD: systematic literature review. Chest. (2013) 144:1163–78. 10.1378/chest.12-2847 23722528

[14] Morgan AD Zakeri R Quint JK. Defining the relationship between COPD and CVD: what are the implications for clinical practice? Ther Adv Respir Dis. (2018) 12:1–16. 10.1177/1753465817750524 29355081 PMC5937157

[15] Kunisaki KM Dransfield MT Anderson JA Brook RD Calverley PMA Celli BR. Exacerbations of chronic obstructive pulmonary disease and cardiac events. Am J Respir Crit Care Med. (2018) 198:51–7. 10.1164/rccm.201711-2239OC 29442524 PMC6913068

[16] Donaldson GC Hurst JR Smith CJ Hubbard RB Wedzicha JA. Increased risk of myocardial infarction and stroke following exacerbation of COPD. Chest. (2010) 137:1091–7. 10.1378/chest.09-2029 20022970

[17] Rothnie KJ Connell O Müllerová H Smeeth L Pearce N Douglas I. Myocardial infarction and ischemic stroke after exacerbations of chronic obstructive pulmonary disease. Ann Am Thorac Soc. (2018) 15:935–46. 10.1513/AnnalsATS.201710-815OC 29723057 PMC6322039

[18] Lahousse L Niemeijer MN van den Berg ME Rijnbeek PR Joos GF Hofman A. Chronic obstructive pulmonary disease and sudden cardiac death: the rotterdam study. Eur Heart J. (2015) 36:1754–61. 10.1093/eurheartj/ehv121 25920404

[19] Sin DD Man SF. Chronic obstructive pulmonary disease as a risk factor for cardiovascular morbidity and mortality. Proc Am Thorac Soc. (2005) 2:8–11. 10.1513/pats.200404-032MS 16113462

[20] Onishi K. Total management of Chronic Obstructive Pulmonary Disease (COPD) as an independent risk factor for cardiovascular disease. J Cardiol. (2017) 70:128–34. 10.1016/j.jjcc.2017.03.001 28325523

[21] van Eeden S Leipsic J Man SF Sin DD. The relationship between lung inflammation and cardiovascular disease. Am J Respir Crit Care Med. (2012) 186:11–6. 10.1164/rccm.201203-0455PP 22538803

[22] Fermont JM Masconi KL Jensen MT Ferrari R Di Lorenzo VA Marott JM. Biomarkers and clinical outcomes in COPD: a systematic review and meta-analysis. Thorax. (2019) 74:439–46. 10.1136/thoraxjnl-2018-211855 30617161 PMC6484697

[23] Chen YW Leung JM Sin DD. A systematic review of diagnostic biomarkers of COPD exacerbation. PLoS ONE. (2016) 11:e0158843. 10.1371/journal.pone.0158843 27434033 PMC4951145

[24] Gonçalves I Guimarães MJ Van Zeller M Menezes F Moita J Simão P. Clinical and molecular markers in COPD. Pulmonology. (2018) 24:250–9. 10.1016/j.pulmoe.2018.02.005 29898875

[25] Adamson PD Anderson JA Brook RD Calverley PMA Celli BR Cowans NJ. Cardiac troponin I and cardiovascular risk in patients with chronic obstructive pulmonary disease. JACC. (2018) 72:1126–37. 10.1016/j.jacc.2018.06.051 30165984 PMC6119211

[26] Zagaceta J Bastarrika G Zulueta JJ Colina I Alcaide AB Campo A. Prospective comparison of non-invasive risk markers of major cardiovascular events in COPD patients. Respir Res. (2017) 18:175. 10.1186/s12931-017-0658-y 28962654 PMC5622585

[27] Bhatt SP Nath HP Kim Y Ramachandran R Watts JR Terry NLJ. Centrilobular emphysema and coronary artery calcification: mediation analysis in the SPIROMICS cohort. Respir Res. (2018) 19:257. 10.1186/s12931-018-0946-1 30563576 PMC6299495

[28] Hackshaw A Morris K Boniface S Tang JL Milenković D. Low cigarette consumption and risk of coronary heart disease and stroke: meta-analysis of 141 cohort studies in 55 study reports. BMJ. (2018) 360:j5855. 10.1136/bmj.j5855 29367388 PMC5781309

[29] Mulpuru S LI L Ye L Hatchette T Andrew MK Ambrose A. Effectiveness of influenza vaccination on hospitalizations and risk factors for severe outcomes in hospitalized patients with COPD. Chest. (2019) 155:69–78. 10.1016/j.chest.2018.10.044 30616737

[30] Lubin JH Couper D Lutsey PL Woodward M Yatsuya H Huxley RR. Risk of cardiovascular disease from cumulative cigarette use and the impact of smoking intensity. Epidemiology. (2016) 27:395–404. 10.1097/EDE.0000000000000437 26745609 PMC5482174

[31] Bhatt SP Kim Y Harrington KF Hokanson Lutz SM Cho MH. Smoking duration alone provides stronger risk estimates of chronic obstructive pulmonary disease than pack-years. BMJ. (2018) 73:414–21. 10.1136/thoraxjnl-2017-210722 29326298 PMC5903957

[32] World Health Organization 7 Million Premature Deaths Annually Linked to Air Pollution. WHO (2014). Available online at: https://www.who.int/mediacentre/news/releases/2014/air-pollution (accessed May 27, 2019).

[33] Pope CA Ezzati M Cannon JB Allen RT Jerrett M Burnett RT Mortality risk and PM2.5 air pollution in the USA: an analysis of a national prospective cohort. Air Qual Atmos Health. (2017) 11:245–52. 10.1007/s11869-017-0535-3

[34] Bartlett J Predazzi IM Williams SM Bush WS Kim Y Havas S. Is isolated low high-density lipoprotein cholesterol a cardiovascular disease risk factor? new insights from the framingham offspring study. Circ Cardiovasc Qual and Outcomes. (2016) 9:206–12. 10.1161/CIRCOUTCOMES.115.002436 27166203 PMC4871717

[35] Tipping RW Ford CE Simpson LM Walldius G Jungner I Folsom AR Lipoprotein(a) concentration and the risk of coronary heart disease, stroke, and nonvascular mortality. JAMA. (2009) 302:412–23. 10.1001/jama.2009.1063 19622820 PMC3272390

[36] Shen Y Yang T Guo S Li X Chen L Wang T. Increased serum Ox-LDL levels correlated with lung function, inflammation, and oxidative stress in COPD. Mediators Inflam. (2013) 2013:972347 10.1155/2013/972347 24078777 PMC3774040

[37] Basili S Ferroni P Vieri M Cardelli P Ceci F Paradiso M. Lipoprotein(a) serum levels in patients affected by chronic obstructive pulmonary disease. Atherosclerosis. (1999) 147:249–52. 10.1016/S0021-9150(99)00192-6 10559510

[38] Chan MC Lin CH Kou YR. Hyperlipidemia in COPD is associated with decreased incidence of pneumonia and mortality: a nationwide health insurance data-based retrospective cohort study. Int J COPD. (2016) 11:1053–9 10.2147/COPD.S102708 27274227 PMC4876799

[39] Baigent C Blackwell L Emberson J Holland LE Reith C Bhala N. Efficacy and safety of more intensive lowering of LDL cholesterol: a meta-analysis of data from 170,000 participants in 26 randomised trials. Lancet. (2010) 376:1670–81. 10.1016/S0140-6736(10)61350-5 21067804 PMC2988224

[40] Peters SA Huxley RR Woodward M. Diabetes as risk factor for incident coronary heart disease in women compared with men: a systematic review and meta-analysis of 64 cohorts including 858,507 individuals and 28,203 coronary events. Diabetologia. (2014) 57:1542–51. 10.1007/s00125-014-3260-6 24859435

[41] Lawes CM Vander H S Rodgers A. Global burden of blood-pressure-related disease, 2001. Lancet. (2008) 371:1513–8. 10.1016/S0140-6736(08)60655-8 18456100

[42] Lewington S Clarke R Qizilbash N Peto R Collins R Prospective Studies Collaboration. Age-specific relevance of usual blood pressure to vascular mortality: a meta-analysis of individual data for one million adults in 61 prospective studies. Lancet. (2002) 360:1903–13. 10.1016/S0140-6736(02)11911-8 12493255

[43] Phrommintikul A Kuanprasert S Wongcharoen W Kanjanavanit R Chaiwarith R Sukonthasarn A. Influenza vaccination reduces cardiovascular events in patients with acute coronary syndrome. Eur Heart J. (2011) 32:1730–5. 10.1093/eurheartj/ehr004 21289042

[44] Vlachopoulos CV Terentes-Printzios DG Aznaouridis KA Pietri PG Stefanadis CI. Association between pneumococcal vaccination and cardiovascular outcomes: a systematic review and meta-analysis of cohort studies. Eur Assoc Prev Cardiol. (2015) 22:1185–99. 10.1177/2047487314549512 25252595

[45] Herath SC Normansell R Maisey S Poole P. Prophylactic antibiotic therapy for chronic obstructive pulmonary disease (COPD). Cochrane Database Syst Rev. (2018) 10:CD009764. 10.1002/14651858.CD009764.pub3 30376188 PMC6517028

[46] Shah AM Pfeffer MA Hartley LH Moy LA Gersh BJ Rutherford JD. Risk of all-cause mortality, recurrent myocardial infarction, and heart failure hospitalization associated with smoking status following myocardial infarction with left ventricular dysfunction. Am J Cardiol. (2010) 106:911–6. 10.1016/j.amjcard.2010.05.021 20854949

[47] Ockene JK Kuller LH Svendsen KH Meilahn E. The relationship of smoking cessation to coronary heart disease and lung cancer in the Multiple Risk Factor Intervention Trial (MRFIT). Am J Public Health. (1990) 80:954–8. 10.2105/AJPH.80.8.954 2368857 PMC1404774

[48] Au DH Bryson CL Chien JW Sun H Udris EM Evans LE. The Effects of smoking cessation on the risk of chronic obstructive pulmonary disease exacerbations. J Gen Intern Med. (2009) 24:457–63. 10.1007/s11606-009-0907-y 19194768 PMC2659150

[49] Thakur M Nuyts PAW Boudewijns EA Kim JF Faber T Babu GR. Impact of improved cookstoves on women's and child health in low and middle income countries: a systematic review and meta-analysis. Thorax. (2018) 73:1026-40. 10.1136/thoraxjnl-2017-210952 29925674

[50] Nathan DM Cleary PA Backlund JY Genuth SM Lachin JM Orchard TJ. Intensive diabetes treatment and cardiovascular disease in patients with type 1 diabetes. N Engl J Med. (2005) 353:2643–53. 10.1056/NEJMoa052187 16371630 PMC2637991

[51] Antonakoudis G Poulimenos L Kifnidis K Zouras C Antonakoudis H. Blood pressure control and cardiovascular risk reduction. Hippokratia. (2007) 11:114–19. 19582204 PMC2658793

[52] Reis AJ Alvez C Furtado S Ferreira J Drummond M Robalo-Cordeiro C. COPD exacerbations: management and hospital discharge. Pulmonology. (2018) 24:345–50. 10.1016/j.pulmoe.2018.06.006 30049647

[53] Li J Sun S Tang R Qiu H Huang Q Mason TG. Major air pollutants and risk of COPD exacerbations: a systematic review and meta-analysis. Int J Chron Obstruct Pulmon. (2016) 11:3079–91. 10.2147/COPD.S122282 28003742 PMC5161337

[54] Lee J Jung HM Kim SK Yoo KH Jung K Lee SH. Factors associated with chronic obstructive pulmonary disease exacerbation, based on big data analysis. Sci Rep. (2019) 9:6679. 10.1038/s41598-019-43167-w 31040338 PMC6491439

[55] Goto T Tsugawa Y Faridi MK Camargo CA Hasegawa K Reduced risk of acute exacerbation of chronic obstructive pulmonary disease after bariatric surgery: a self-controlled case series study. Chest. (2018) 153:611–7. 10.1016/j.chest.2017.07.003 28716643

[56] Montserrat-Capdevila J Godoy P Marsal JR Barbé F Galván L. Risk factors for exacerbation in chronic obstructive pulmonary disease: a prospective study. Int J Tuberc Lung Dis. (2016) 20:389–95. 10.5588/ijtld.15.0441 27046722

[57] Hewitt R Farne H Ritchie A Luke E Johnston SL Mallia P. The role of viral infections in exacerbations of chronic obstructive pulmonary disease and asthma. Ther Adv Respir Dis. (2016) 10:158–74. 10.1177/1753465815618113 26611907 PMC5933560

[58] Biancardi E Fennell M Rawlinson W Thomas PS. Viruses are frequently present as the infecting agent in acute exacerbations of chronic obstructive pulmonary disease in patients presenting to hospital. Internal Med J. (2016) 46:1160–5. 10.1111/imj.13213 27515577 PMC7165870

[59] Boixeda R Almagro P Díez-Manglano J Cabrera FJ Recio J Martin-Garrido I. Bacterial flora in the sputum and comorbidity in patients with acute exacerbations of COPD. Int J Chron Obstruct Pulmon Dis. (2015) 10:2581–91. 10.2147/COPD.S88702 26664106 PMC4671781

[60] Choi J Oh JY Lee YS Hur GY Lee SY Shim JJ. Bacterial and viral identification rate in acute exacerbation of chronic obstructive pulmonary disease in Korea. Yonsei Med J. (2019) 60:216–22. 10.3349/ymj.2019.60.2.216 30666844 PMC6342712

[61] Kwong JC Schwartz KL Campitelli MA Chung H Crowcroft NS Karnauchow T Acute myocardial infarction after laboratory-confirmed influenza infection. N Engl J Med. (2018) 378:345–53. 10.1056/NEJMoa1702090 29365305

[62] Musher DM Abers MS Corrales-Medina VF Acute infection and myocardial infarction. N Engl J Med. (2019) 380:171–6. 10.1056/NEJMra1808137 30625066

[63] Madjid M Vela D Khalili-Tabrizi H Casscells SW Litovsky S. Systemic infections cause exaggerated local inflammation in atherosclerotic coronary arteries: clues to the triggering effect of acute infections on acute coronary syndromes. Tex Heart Inst J. (2007) 34:11–8. 17420787 PMC1847934

[64] Jaw JE Tsuruta M Oh Y Schipilow J Hirano Y Ngan DA. Lung exposure to lipopolysaccharide causes atherosclerotic plaque destabilisation. Eur Respir J. (2016) 48:205–15. 10.1183/13993003.00972-2015 27009170

[65] Center for disease Control and Prevention National Smoking and Tobacco Use Statistics Report: Tobacco-Related Mortality. Atlanta, GA: US Department of Health and Human Services (2014). Available online at: https://www.cdc.gov/tobacco/data_statistics (accessed May 27, 2019).

[66] U.S. Department of Health and Human Services. How Tobacco Smoke Causes Disease: The Biology and Behavioral Basis for Smoking-Attributable Disease: A Report of the Surgeon General. Atlanta, GA: U.S. Department of Health and Human Services, Centers for Disease Control and Prevention, National Center for Chronic Disease Prevention and Health Promotion, Office on Smoking and Health (2010). Available online at: https://www.hhs.gov/sites/default/files/consequences-smoking-exec-summary.pdf (accessed May 27, 2019). 21452462

[67] Isajevs S Taivans I Strazda G Kopeika U. Decreased FOXP3 expression in small airways of smokers with COPD. Eur Respir J. (2009) 33:61–7. 10.1183/09031936.00145307 18684844

[68] Bhat TA Panzica L Kalathil SG Thanavala Y. Immune dysfunction in patients with chronic obstructive pulmonary disease. Ann Am Thor Soc. (2015) 12(Suppl. 2):S169–75. 10.1513/AnnalsATS.201503-126AW 26595735 PMC4722840

[69] Nielsen VG Hafner DT Steinbrenner EB. Tobacco smoke-induced hypercoagulation in human plasma: role of carbon monoxide. Blood Coagul Fibrinolysis. (2013) 24:405–10. 10.1097/MBC.0b013e32835d5458 23429254

[70] Burke A FitzGerald GA. Oxidative stress and smoking-induced vascular injury. Progr Cardiovasc Dis. (2003) 46:79–90. 10.1016/S0033-0620(03)00076-8 12920701

[71] Josephs L Culliford D Johnson M Thomas M. Improved outcomes in ex-smokers with COPD: a UK primary care observational cohort study. Eur Respir J. (2017) 49:1602114. 10.1183/13993003.02114-2016 28536250 PMC5460640

[72] Pelkonen M Notkola I Tukiainen H Tervahauta M Tuomilehto J Nissinen A. Smoking cessation, decline in pulmonary function and total mortality: a 30 year follow up study among the finnish cohorts of the seven countries study. Thorax. (2001) 56:703–7. 10.1136/thorax.56.9.703 11514691 PMC1746131

[73] Song C He J Wu L Jin T Chen X Li R. Health burden attributable to ambient PM2.5 in China. Environ Pollut. (2017) 223:575–86. 10.1016/j.envpol.2017.01.060 28169071

[74] Mills NL Törnqvist H Gonzalez MC Vink E Robinson SD Söderberg S. Ischemic and thrombotic effects of dilute diesel-exhaust inhalation in men with coronary heart disease. N Engl J Med. (2007) 357:1075–82. 10.1056/NEJMoa066314 17855668

[75] Suwa T Hogg JC Quinlan KB Ohgami A Vincent R van Eeden SF. Particulate air pollution induces progression of atherosclerosis. J Am Coll Cardiol. (2002) 39:935–42. 10.1016/S0735-1097(02)01715-1 11897432

[76] Kido T Tamagawa E Bai N Suda K Yang HH Li Y. Particulate matter induces translocation of IL-6 from the lung to the systemic circulation. Am J Respir Cell Mol Biol. (2011) 44:197–204. 10.1165/rcmb.2009-0427OC 20378751

[77] Nordestgaard BG Langsted A. Lipoprotein (a) as a cause of cardiovascular disease: insights from epidemiology, genetics, and biology. J Lipid Res. (2016) 57:1953–75. 10.1194/jlr.R071233 27677946 PMC5087876

[78] Saeed A Feofanova EV Yu B Sun W Virani SS Nambi V. Remnant-like particle cholesterol, low-density lipoprotein triglycerides, and incident cardiovascular disease. J Am Coll Cardiol. (2018) 72:156–69. 10.1016/j.jacc.2018.04.050 29976289 PMC6051722

[79] Boekholdt SM Arsenault BJ Mora S Pedersen TR LaRosa JC Nestel PJ. Association of LDL cholesterol, non-HDL cholesterol, and apolipoprotein B levels with risk of cardiovascular events among patients treated with statins: a meta-analysis. JAMA. (2012) 307:1302–9. 10.1001/jama.2012.3888 22453571

[80] Zafirova-Ivanovska B Stojkovikj J Dokikj D Anastasova S Debresliovska A Zejnel S. The level of cholesterol in COPD patients with severe and very severe stage of the disease. Maced J Med Sci. (2016) 4:277–82. 10.3889/oamjms.2016.063 27335600 PMC4908745

[81] Mazière C Mazière J. Activation of transcription factors and gene expression by oxidized low-density lipoprotein. Free Rad Biol Med. (2009) 46:127–37. 10.1016/j.freeradbiomed.2008.10.024 18996472

[82] Sedgwick JB Hwang YS Gerbyshak HA Kita H Busse WW. Oxidized low-density lipoprotein activates migration and degranulation of human granulocytes. Am J Respir Cell Mol Biol. (2003) 29:702–9. 10.1165/rcmb.2002-0257OC 12777245

[83] Hunninghake DB. Cardiovascular disease in chronic obstructive pulmonary disease. Proc Am Thor Soc. (2005) 2:44–9. 10.1513/pats.200410-050SF 16113468

[84] Batt KV Patel L Botham KM Suckling KE. Chylomicron remnants and oxidised low density lipoprotein have differential effects on the expression of mRNA for genes involved in human macrophage foam cell formation. J Mol Med. (2004) 82:449–58. 10.1007/s00109-004-0551-2 15156288

[85] Chen H Li Z Dong L Wu Y Shen H Chen Z. Lipid metabolism in chronic obstructive pulmonary disease. Int J Chronic Obstruct Pulmon Dis. (2019) 14:1009–18. 10.2147/COPD.S196210 31190786 PMC6524761

[86] Cohen JC Boerwinkle E Mosley TH Hobbs HH. Sequence variations in PCSK9, low LDL, and protection against coronary heart disease. N Engl J Med. (2006) 354:1264–72. 10.1056/NEJMoa054013 16554528

[87] Fan W Epidemiology in diabetes mellitus and cardiovascular disease. Cardiovasc Endocrinol Metab. (2017) 6:8–16. 10.1097/XCE.0000000000000116 PMC6768526 31646113

[88] Morrish NJ Wang SL Stevens LK Fuller JH Keen H. Mortality and causes of death in the WHO multinational study of vascular disease in diabetes. Diabetologia. (2001) 44(Suppl. 2):S14–21. 10.1007/PL00002934 11587045

[89] Peters SA Huxley RR Woodward M. Diabetes as a risk factor for stroke in women compared with men: a systematic review and meta-analysis of 64 cohorts, including 775,385 individuals and 12,539 strokes. Lancet. (2014) 383:1973–80. 10.1016/S0140-6736(14)60040-4 24613026

[90] Davis W Knuiman M Kendall P Grange V Davis TME. Glycemic exposure is associated with reduced pulmonary function in type 2 diabetes: the fremantle diabetes study. Diabetes Care. (2004) 27:752–7. 10.2337/diacare.27.3.752 14988297

[91] Aneja A Tang WHW Bansilal S Garcia MJ Farkouh ME. Diabetic cardiomyopathy: insights into pathogenesis, diagnostic challenges, and therapeutic options. Am J Med. (2008) 121:748–57. 10.1016/j.amjmed.2008.03.046 18724960

[92] Cazzola M Calzetta L Rogliani P Lauro D Novelli L Page CP. High glucose enhances responsiveness of human airways smooth muscle via the Rho/ROCK pathway. Am J Respir Cell Mol Biol. (2012) 47:509–16. 10.1165/rcmb.2011-0449OC 22652200

[93] Fernandes LB Henry PJ Goldie RG. Rho kinase as a therapeutic target in the treatment of asthma and chronic obstructive pulmonary disease. Ther Adv Res Disease. (2007) 1:25–33. 10.1177/1753465807080740 19124345

[94] Rayner LH McGovern AP Sherlock J Gatenby P Correa A Creagh-Brown B. Type 2 diabetes: a protective factor for COPD? Primary Care Diabetes. (2018) 12:438–44. 10.1016/j.pcd.2018.05.002 29843977

[95] Weaver DJ Mitsnefes MM Effects of systemic hypertension on the cardiovascular system. Progr Pediatr Cardiol. (2016) 41:59–65. 10.1016/j.ppedcard.2015.11.005

[96] Huang TJ Bolton E Miller E Tal-Singer R Rabinovich RA Palmer CNA. Age-dependent elastin degradation is enhanced in chronic obstructive pulmonary disease. Eur Respir J. (2016) 48:1215–8. 10.1183/13993003.01125-2016 27587547

[97] Sarkar M Chronic obstructive pulmonary disease and arterial stiffness. EMJ Respir. (2016) 4:114–21.

[98] Nguyen D Cat A Montezano AC Burger D Touyz RM Angiotensin II, NADPH oxidase, and redox signaling in the vasculature. Antioxid Redox Signal. (2013) 19:1110-20. 10.1089/ars.2012.4641 22530599 PMC3771549

[99] Mannam P Srivastava A Sugunaraj JP Lee PJ Sauler M. Oxidants in acute and chronic lung disease. J Blood Lymph. (2014) 4:1000128. 10.4172/2165-7831.1000128 25705575 PMC4335304

[100] Upadhya B Rocco M Lewis CE Oparil S Lovato LC Cushman WC. Effect of intensive blood pressure treatment on heart failure events in the systolic blood pressure reduction intervention trial. Circ Heart Fail. (2017) 10:e003613. 10.1161/CIRCHEARTFAILURE.116.003613 28364091 PMC5384646

[101] Sprint Research Group Wright JT Jr Williamson JD Whelton PK Snyder JK Sink KM. A Randomized trial of intensive versus standard blood-pressure control. N Engl J Med. (2015) 373:2103–16. 10.1056/NEJMoa1511939 26551272 PMC4689591

[102] Vaes AW Garcia-Aymerich J Marott JL Benet M Groenen MTJ Schnohr P. Changes in physical activity and all-cause mortality in COPD. Eur Respir J. (2014) 44:1199–209. 10.1183/09031936.00023214 25063247

[103] Esteban C Arostegui I Aburto M Moraza J Quintana JM Aizpiri S. Influence of changes in physical activity on frequency of hospitalizationin chronic obstructive pulmonary disease. Respirology. (2014) 19:330–8. 10.1111/resp.12239 24483954

[104] Ryrsø CK Godtfredsen NS Kofod LM Lavesen M Mogensen L Tobberup R. Lower mortality after early supervised pulmonary rehabilitation following COPD-exacerbations: a systematic review and meta-analysis. BMC Pulmon Med. (2018) 18:154. 10.1186/s12890-018-0718-1 30219047 PMC6139159

[105] Du Q Sun Y Ding N Lu L Chen Y. Beta-blockers reduced the risk of mortality and exacerbation in patients with COPD: a meta-analysis of observational studies. PLoS ONE. (2014) 9:e113048. 10.1371/journal.pone.0113048 25427000 PMC4245088

[106] Dransfield MT McAllister DA Anderson JA Brook RD Calverley PMA Celli BR B-blocker therapy and clinical outcomes in patients with moderate chronic obstructive pulmonary disease and heightened cardiovascular risk an observational substudy of SUMMIT. Ann Am Thor Soc. (2018) 15:608–14. 10.1513/AnnalsATS.201708-626OC 29406772

[107] Gershon A Croxford R Calzavara A To T Stanbrook MB Upshur R. Cardiovascular safety of inhaled long-acting bronchodilators in individuals with chronic obstructive pulmonary disease. JAMA Intern Med. (2013) 173:1175–85. 10.1001/jamainternmed.2013.1016 23689820

[108] Singh S Loke YK Furberg CD. Inhaled anticholinergics and risk of major adverse cardiovascular events in patients with chronic obstructive pulmonary disease: a systematic review and meta-analysis. JAMA. (2008) 300:1439–50. 10.1001/jama.300.12.1439 18812535

[109] Dong YH Lin HH Shau WY Wu YC Chang CH Lai MS. Comparative safety of inhaled medications in patients with chronic obstructive pulmonary disease: systematic review and mixed treatment comparison meta-analysis of randomised controlled trials. Thorax. (2013) 68:48–56. 10.1136/thoraxjnl-2012-201926 23042705

[110] Bradbury P Traini D Ammnit AJ Young PM Ong HX. Repurposing of statins via inhalation to treat lung inflammatory conditions. Adv Drug Delivery Rev. (2018) 133:93–106. 10.1016/j.addr.2018.06.005 29890243

[111] Cao C Wu Y Xu Z Lv D Zhang C Lai T. The effect of statins on chronic obstructive pulmonary disease exacerbation and mortality: a systematic review and meta-analysis of observational research. Sci Rep. (2015) 5:16461. 10.1038/srep16461 26553965 PMC4639730

[112] Criner GJ Connett JE Aaron SD Albert RK Bailey WC Casaburi R. Simvastatin for the prevention of exacerbations in moderate-to-severe COPD. N Engl J Med. (2014) 370:2201–10. 10.1056/NEJMoa1403086 24836125 PMC4375247

